# Does the High Prevalence of Vitamin D Deficiency in African Americans Contribute to Health Disparities?

**DOI:** 10.3390/nu13020499

**Published:** 2021-02-03

**Authors:** Bruce N. Ames, William B. Grant, Walter C. Willett

**Affiliations:** 1 Molecular and Cell Biology, Emeritus, University of California, Berkeley, CA 94720, USA; bnames@berkeley.edu; 2 Sunlight, Nutrition and Health Research Center, San Francisco, CA 94164-1603, USA; 3 Departments of Nutrition and Epidemiology, Harvard T.H. Chan School of Public Health, Boston, MA 02115, USA; wwillett@hsph.harvard.edu; 4 Channing Division of Network Medicine, Department of Medicine, Brigham and Women’s Hospital, Harvard Medical School, Boston, MA 02115, USA

**Keywords:** African American, Hispanic, European American, blacks, whites, health disparities, vitamin D, 25-hydroxyvitamin D, UVB

## Abstract

African Americans have higher incidence of, and mortality from, many health-related problems than European Americans. They also have a 15 to 20-fold higher prevalence of severe vitamin D deficiency. Here we summarize evidence that: (i) this health disparity is partly due to insufficient vitamin D production, caused by melanin in the skin blocking the UVB solar radiation necessary for its synthesis; (ii) the vitamin D insufficiency is exacerbated at high latitudes because of the combination of dark skin color with lower UVB radiation levels; and (iii) the health of individuals with dark skin can be markedly improved by correcting deficiency and achieving an optimal vitamin D status, as could be obtained by supplementation and/or fortification. Moderate-to-strong evidence exists that high 25-hydroxyvitamin D levels and/or vitamin D supplementation reduces risk for many adverse health outcomes including all-cause mortality rate, adverse pregnancy and birth outcomes, cancer, diabetes mellitus, Alzheimer’s disease and dementia, multiple sclerosis, acute respiratory tract infections, COVID-19, asthma exacerbations, rickets, and osteomalacia. We suggest that people with low vitamin D status, which would include most people with dark skin living at high latitudes, along with their health care provider, consider taking vitamin D_3_ supplements to raise serum 25-hydroxyvitamin D levels to 30 ng/mL (75 nmol/L) or possibly higher.

## 1. Introduction

The vitamin D hormone controls the activity of thousands of protein-encoding human genes [1]. Therefore, optimum levels are likely to be important for health. Synthesis of vitamin D in human skin depends on solar UVB radiation, whose levels are low at high latitudes, as in the United States and Europe, and highest at equatorial latitudes [2]. High concentration of melanin, the brown-black pigment in skin, is appropriate for the high UV radiation dose regions such as in the tropical plains as it absorbs UVB radiation absorption, thereby reducing production of free radicals and destruction of folate, but permitting adequate vitamin D production [3]. At higher latitudes, where the UVB radiation dose is lower [2], the rate of synthesis of vitamin D correspondingly decreases, potentially disrupting many metabolic functions that depend on that vitamin and leading to poorer health.

Here we discuss various aspects of such latitude–skin color mismatch and health disparities. By latitude–skin color mismatch, we mean that skin pigmentation is not appropriate for the solar UV doses at various latitudes, either too dark as for African Americans (defined as people living in the United States with some African ancestry) to efficiently produce vitamin D, or too light to protect against the harmful effects of UV radiation as for people living close to the equator, such as those with Anglo-Celtic ancestry in Australia. (Note that people of African descent have dark melanin, called eumelanin, while Anglo-Celtics have yellow-to-reddish melanin called pheomelanin.) This mismatch is particularly impactful in African Americans, whose dark skin is well adapted to the high UVB levels at low equatorial latitudes [3]. However, as a legacy of slavery and more recent migration, African Americans now reside at higher latitudes than in their ancestral environments. That geographic shift is largely responsible for a high prevalence of vitamin D deficiency (serum 25-hydroxyvitamin D (25(OH)D) levels < 20 ng/mL) in African Americans [4,5] independent of diet and other factors [6]. This high prevalence of deficiency potentially contributes to many health disparities. Because vitamin D deficiency can be easily remedied by supplementation or, to a lesser extent, fortification of food, the health implications of this deficiency are important to understand.

Many factors adversely affect the health of African Americans, including high rates of poverty [7], poor housing and residential environments [8], and lack of access to affordable health care. Living in racially segregated, poor neighborhoods also exposes residents to risk of crime [9], thereby limiting time spent outdoors, as well as reducing access to well-stocked grocery stores and pharmacies. Limited educational opportunities frequently result in having jobs with high social interaction and thus greater risk of COVID-19. The high incarceration rate of African American males has resulted in many children being raised by single mothers. While these factors play important roles in racial health disparities and require sustained efforts to correct at individual and societal levels, vitamin D deficiency can be corrected rapidly and inexpensively. In this review we examine the potential health benefits of addressing this deficiency.

This narrative review considers the potential health effects of inadequate vitamin D in humans. Although the motivation for this review is the high prevalence of vitamin D deficiency in African Americans, we draw on the literature from all populations because our underlying biology is similar across all racial groups, even though the prevalence of exposures, here serum levels of 25(OH)D, can differ greatly. Thus, the findings have implications for other groups with darker skin and low 25(OH)D levels, such as US immigrants from Mexico and South Asia, and for European Americans with limited sun exposure. (The term European Americans is used to represent white, non-Hispanic Americans.) 

When they are available, we cite meta-analyses or pooled primary data from multiple studies. Because ideal randomized trials are often difficult or impossible to conduct, conclusions regarding causality will usually need to be based on the weight of evidence from multiple types of study [10]. The strengths and limitations of the various approaches to determine relationships between vitamin D and health outcomes are presented in the Appendix A. Ideally, vitamin D’s health effects in populations with dark skin would be evaluated directly in such groups, but in most studies the number of such participants has been too small to evaluate separately. Nevertheless, we highlight studies of subgroups of African Americans and Hispanics (17% of the U.S. population) when available. We also pay special attention to subgroups with low baseline serum levels of vitamin D in randomized trials as this where an effect of supplementation may be expected to be seen; failure to do this may lead to misleading negative conclusions. In some randomized trials comparing vitamin D supplements with a placebo, those with low serum levels of 25(OH)D are excluded for ethical reasons and/or are treated, again potentially leading to misleading conclusions.

## 2. Current Status of Knowledge

### 2.1. Vitamin D: Synthesis and Metabolism

Vitamin D_3_ is synthesized in human skin by the UVB-dependent conversion of 7-dehydrocholesterol to vitamin D_3_ (herein referred to as vitamin D when used as a supplement). Vitamin D_3_ is then converted to 25(OH)D_3_, a precursor of the crucial vitamin D steroid hormone, 1,25-dihydroxyvitamin D_3_, or calcitriol, in a reaction requiring magnesium [11,12], which is widely deficient in the American diet [13]. Calcitriol binds to a specific binding protein, the vitamin D receptor (VDR). The resulting complex interacts with human DNA regulatory sequences known as vitamin D response elements (VDREs; 15 bases long), which reportedly vary in number between a few thousand to ten thousand [14,15]. VDREs respond specifically to calcitriol by activating or inactivating their adjacent genes [16,17,18]; this response may vary depending on the location of the VDRE and level of 25(OH)D [15]. The unusually large number of calcitriol-responsive DNA sites strongly suggests that sufficiently high 25(OH)D levels, which may vary by outcome are necessary for optimal health and longevity [16,19].

### 2.2. Evolution of Skin Pigmentation

**Figure 1:**
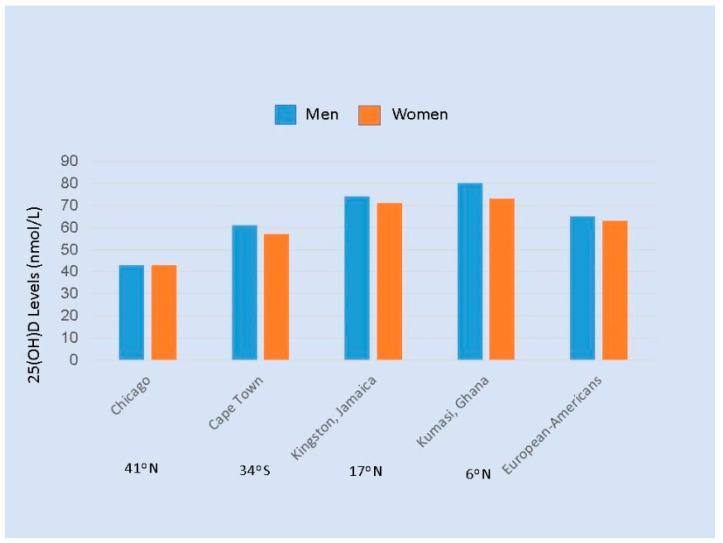
Average serum 25(OH)D levels (nmol/L) in men and women of African Ancestry ages 25 to 45 years living in four sites [32], and European Americans. The latitudes of the cities are given below the names of the cities. Note: Divide by 2.5 to convert nmol/L to ng/mL.

Skin pigmentation is an evolutionary response to the intense solar UVB at low latitudes, where early humans evolved. Dark skin, through the presence of abundant melanin, protected humans living in Africa, southern India, and other parts of Asia against strong UVB, which causes severe sunburn, damages DNA, and destroys skin folate [3,20,21,22]. According to a widely accepted hypothesis, people in ancient times moved from low to higher latitudes, and skin pigment evolved (by several mutations) to be lighter, depending on distance from the equator, permitting more efficient production of vitamin D [3,23,24,25,26]. Others have suggested that lighter skin resulted from the acquisition of genetic variants from populations that immigrated into northern Europe, but this is still compatible with production of vitamin D being the initial selective factor for these variants [27]. (These authors also hypothesize that variations in genes encoding for proteins responsible for the transport, metabolism and signaling of vitamin D provide alternative mechanisms of adaptation to a life in northern latitudes without suffering the consequences of vitamin D deficiency. However, such mechanisms and loss of melanin are not mutually exclusive, and in either case they would leave people of African descent now living in northern latitudes at risk of vitamin D deficiency.) The importance of solar exposure is illustrated by findings that Africans with dark skin living at low latitudes have levels of 25(OH)D of 29 to 46 ng/mL [28,29,30], that are substantially higher than those of African Americans (mean 25(OH)D ~16 ng/mL) [31]. These differences, and the similarity in levels of 25(OH)D in European Americans and Africans living in Africa, are shown in Figure 1 [32]. Thus, darker skin pigmentation in Africans living in Africa appears to allow adequate vitamin D synthesis while protecting against sunburn and other damage. Direct genetic evidence that melanin reduces synthesis of vitamin D is provided by findings that Nigerians with albinism have significantly higher 25(OH)D levels than those with normal pigmentation [33]. The interaction between skin melanin and sunlight was further illustrated in a study of pregnant women in the southeastern US; the ratios of winter-to-summer prevalence of vitamin D insufficiency were 3.58 (95% CI 1.64 to 7.81) for European-American, 1.52 (95% CI 1.18 to 1.95) for Hispanic, and 1.14 (95% CI 0.99 to 1.30) for African-American women [34].

In contrast to the slow migration in ancient times, in more recent times there was rapid movement of equatorial Africans to various regions, such as North America, due to slave transport. When the destination is at higher northern latitudes than that of the ancestral country of origin, a mismatch between skin color and UV radiation occurs and lower UVB penetration of the skin to the layer with 7-dehydrocholesterol results in deficient endogenous vitamin D production. The consequent health problems can take years to manifest and thus are both subtle and insidious. A reverse mismatch occurs when light-skinned individuals move to low latitudes (e.g., an Irish person moving to Australia), resulting in increased risk for severe sunburn (and later, high rates of skin cancer). The reverse mismatch is recognized quickly and can be mitigated by using hats and sunscreen.

### 2.3. Prevalence of 25(OH)D Deficiency by Race/ethnicity Group

**Figure 2:**
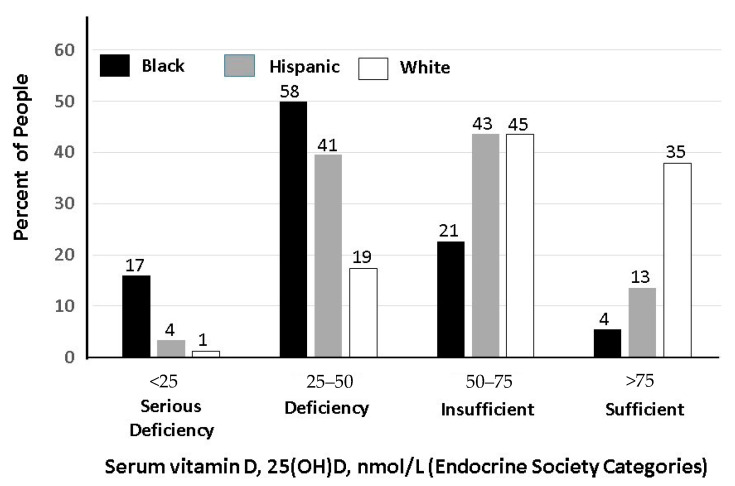
Prevalence of serum 25(OH)D levels in National Health and Nutrition Examination Survey (NHANES) survey, 2001–2010, by race/ethnicity category among nonusers of vitamin D supplements [35]. Additional calculations courtesy of X. Liu. Note: Divide by 2.5 to convert nmol/L to ng/mL.

According to data from National Health and Nutrition Examination Survey (NHANES) 2001–2010, the prevalence of vitamin D deficiency (25(OH)D < 20 ng/mL) among those not taking vitamin D supplements was 75% for non-Hispanic blacks, 44% for Hispanics, and 20% for non-Hispanic whites (Figure 2), whereas severe deficiency (<10 ng/mL) was 17% in non-Hispanic blacks and only 1% in non-Hispanic whites [35]. Although definitions vary [36], there is consensus that levels below 10 ng/mL are a serious concern. A high prevalence of low 25(OH)D levels has also been documented in many other parts of the world [37,38,39,40,41]. 

### 2.4. Vitamin D and Health Outcomes

Most epidemiologic studies of vitamin D and health outcomes have used plasma or serum levels of 25(OH)D to measure vitamin D status. That approach has the advantage of integrating intake, solar exposure, skin color, and genetic factors. A single measure of 25(OH)D serves as a good measure of long-term status for an individual; however, the within-person correlation between 25(OH)D levels decreases as follow-up time increases [42]. Downstream metabolites of 25(OH)D are too variable over time to serve as a stable indicator of vitamin D status [43]. Other indicators of vitamin D status, such as parathyroid hormone, may improve our assessment [44], but have not yet been widely used in epidemiologic studies. Some studies have used vitamin D intake calculated from food intake, with or without supplements.

### 2.5. Skeletal Health

Adequate vitamin D has long been recognized as essential for bone health, and the 2011 Institute of Medicine (IOM) review of vitamin D requirements concluded that rickets and osteomalacia were the only established consequences of low vitamin D status [36]. Thus, the relation to osteomalacia served to set recommendations for vitamin D intake: the estimated average requirement (EAR—at which half the population is deficient and half is not) for serum 25(OH)D was set at 16 ng/mL. On this basis also, levels below 12 ng/mL were considered deficient, 12 to 20 ng/mL were considered “at risk of inadequacy”, and levels above 20 ng/mL were considered sufficient for 97% of the population. Other groups have defined deficiency as levels below 30 ng/mL [45]. Since 2011, much additional evidence has supported the important effects of vitamin D beyond bone health, and the relation between serum levels of 25(OH)D and these health outcomes cannot be assumed to be the same as that with osteomalacia. 

Serum levels of 25(OH)D are positively associated with bone mineral density in both European Americans and African Americans [46], but Africans and African Americans have long been known to have higher bone mineral density (BMD) [47] and lower risk of fragility fractures than Europeans [48]. Possible mechanisms may be that African Americans have higher calcium retention, lower calcium excretion, and greater bone resistance to parathyroid hormone than European Americans [47,49,50]. The reason why populations migrating from Africa to higher latitudes evolved to have weaker bones is unclear, but in the context of low UV radiation a trade-off for reductions in pelvic deformity and obstructed labor has been suggested [51]. Whatever the mechanisms, the greater bone strength of African Americans, and the assumption that the only consequence of low 25(OH)D levels is poor bone health, seems to have led many to believe that the low serum levels 25(OH)D in African Americans are not a concern. Notably, the 2011 IOM review of vitamin D did not emphasize the high prevalence of vitamin D deficiency in African Americans, even by their strictest definition of less than 12 ng/mL, and concluded that “requirements are being met by most if not all persons in both countries [US and Canada]”.

A finding of low levels of vitamin D-binding protein (VDBP) in African Americans, and thus presumably higher biologically active vitamin D, has been suggested as an explanation for healthy bone mass in African Americans despite low 25(OH(D level [52]. However, the report of low VDBP levels was subsequently shown to be an artifact of the monoclonal antibody assay used in that study; when measured by a polyclonal method or proteomic assay, levels of VDBP were similar in African- and European Americans [53]. This, and findings of much higher 25(OH)D levels in Africans living traditional lifestyles in equatorial regions, support the conclusion that the low levels of African Americans are not “natural” but due to environmental factors, primarily inadequate sun exposure.

### 2.6. Pregnancy and Early Development

Pregnant African-American women have higher risk of many pregnancy-related complications than European American or Hispanic women (Table 1). 

Pregnant women with darker skin color have lower 25(OH)D levels than women with lighter skin [34,58,59,60]. Indirect support for a role of vitamin D in development is provided by the finding that during pregnancy, maternal serum levels of 1,25(OH)_2_D increase by 75% and those of 25(OH)D by about 30% [61]. The placenta plays a major role regarding these increases [62]. Further, when 1,25(OH)_2_D stimulates the vitamin D receptors, it can affect the expression of hundreds to thousands of genes [1,15], and fetal development is guided by gene expression.

1. Preeclampsia. In a meta-analysis of data from 27 randomized controlled trials (RCTs) including 4777 participants, vitamin D treatment reduced risk of preeclampsia by 63% (OR = 0.37 (95% CI, 0.26 to 0.52)) [63]. Results were similar with respect to beginning of supplementation, supplementation until delivery, whether or not calcium was also supplemented, and whether the trial was blinded. Increased vitamin D dosage up to 7000 IU/d was associated with reduced risk of preeclampsia.
2. Low birth weight and small for gestational age. In a meta-analysis of 24 RCTs involving 5405 participants, vitamin D supplementation (800 to 7000 IU/day) during pregnancy reduced risk of offspring being small for gestational age by 28% (Risk ratio = 0.72 (95% CI, 0.52 to 0.99, *p* = 0.04)) [64]. In an observational study conducted in Cincinnati involving 276 black infants and 162 white infants, cord blood vitamin D deficiency was associated with being small for gestational age for black infants (OR = 2.4 (95% CI, 1.0 to 5.8, *p* = 0.04)) but not white infants (OR = 1.1 (95% CI, 0.3 to 3.9, *p* = 0.86)) [65]. Vitamin D deficiency was associated with increased risk of preeclampsia among both black and white women: for blacks, OR = 2.3 (95% CI, 1.0 to 5.4, *p* = 0.04) and for whites, OR = 4.1 (95% CI = 1.0, 16.1, *p* = 0.05),
3. Preterm birth. In a meta-analysis of 18 observational studies, maternal 25(OH)D level <20 ng/mL versus >20 ng/mL was associated with a pooled OR = 1.25 (95%CI: 1.13 to 1.38) of preterm delivery [66]. In five studies, this was also the case for spontaneous preterm delivery; for 25(OH)D < 20 ng/mL vs. >20 ng/mL pooled OR = 1.45 (95% CI, 1.20 to 1.75). In a meta-analysis of six RCTs, involving 1880 participants with a total of 77 preterm births, vitamin D supplementation reduced preterm delivery by 43%; the pooled relative risk was 0.57 (95%CI: 0.36 to 0.91)) [66]. The vitamin D dose varied from 400 IU/d to 4000 IU/d. An open-label vitamin D supplementation study involving 1064 pregnant women including African-American and Hispanic and European-American women was conducted in South Carolina [67]. Women were counseled during their first prenatal visit on how to achieve >40 ng/mL 25(OH)D and given free bottles of 5000 IU vitamin D_3_. In the fully-adjusted model, achieving >40 ng/mL vs. <20 ng/mL resulted in an OR for preterm delivery of 0.41 (95% CI, 0.24 to 0.72). Significantly lower risks of preterm birth were seen for both white and non-white women.
4. Neurologic development. In 2008, vitamin D deficiency during pregnancy was hypothesized to be a risk factor for autism [68]. In a meta-analysis of 25 observational studies, higher vs. lower serum levels of 25(OH)D during pregnancy or in newborn blood at birth were associated with a 28% lower risk of attention deficit–hyperactivity disorder in the offspring [pooled relative risk = 0.72 (95% CI = 0.59 go 0.89, *p* = 0.002)] and a 58% lower risk of autism-related traits (pooled relative risk = 0.42 (95% CI = 0.25 to 0.71, *p* = 0.001)) [69]. Vitamin D supplementation during pregnancy has reduced risk of abnormal neurologic development, and administration of 4000 IU/d during pregnancy caused no adverse effects in a trial conducted in South Carolina [61,70].
5. Cesarean delivery. In an observational study in Boston involving 253 women of whom 43 had a cesarean delivery, women with serum 25(OH)D levels lower than 15 ng/mL at time of delivery had 3.8 times the rate of primary cesarean delivery as compared to women with higher levels [71].
6. The overall evidence strongly supports the harmful nature of vitamin D deficiency among pregnant women for both pregnancy-related outcomes and for fetal development. Further, vitamin D supplementation has reduced the risk of pregnancy-related complications, particularly for women with severe deficiency [72].

### 2.7. Cancer

For many cancers, African Americans have higher incidence and mortality rates than European Americans; disparities exist for cancers of the bladder, breast, colon, endometrium, lung, ovary, pancreas, prostate, rectum, testes, and vagina, and for Hodgkin’s lymphoma [73,74] (Table 2). Higher incidence and lower survival both contribute to some of those differences; for 2008–2012, African American males had a 12% higher overall cancer incidence and a 27% higher mortality rate than white men, whereas African American females had a 4% lower incidence rate but a 14% higher mortality rate than white women [74]. In many of the analyses, these differences in cancer rates were adjusted for a variety of potential confounding variables. Smoking, a major cause of cancer, does not account for the disparities because smoking rates for African Americans and European Americans are similar [74].

In single-country geographical ecological studies, solar UVB doses are inversely associated with mortality rates for many cancers among white people [75], and within the U.S. similar inverse associations are seen among both European Americans and African Americans [76,77,78]. Variables related to socioeconomic status can be hard to account for completely, especially in ecological studies. However, among male health professionals with similar education and occupation, African Americans with few risk factors for hypovitaminosis D had risks of cancer similar to those of white men; in contrast, African-American men with several risk factors for hypovitaminosis D had a 57% higher total cancer incidence and 127% higher cancer mortality rate [79]. Risk factors for hypovitaminosis D in this population included living in a region with low solar UVB doses, not spending much recreational time out of doors, and not taking vitamin D supplements. The excess risks were greater for digestive-tract cancers. The mechanisms by which vitamin D may reduce risk of cancer incidence and death include effects on cellular differentiation, proliferation, and apoptosis; anti-angiogenesis; and anti-metastasis [80], as well as anti-inflammatory [80,81] and immune-enhancing [82] mechanisms.

Colorectal cancer. Among various malignancies, low vitamin D status has been most consistently associated with colorectal cancer. In ecological analyses within the United States, colorectal cancer (CRC) mortality among European Americans has been lowest in southwestern states and highest in northeastern states, and lowest in the southern states and highest in the northern states for African Americans (data missing for many states) consistent with the pattern of solar UVB doses in summer [77,78,83]. In an analysis of race and 25(OHD) levels in relation to risk of death due to colorectal cancer [84], a significant two-fold increase in risk was seen among both non-Hispanic white and non-Hispanic black participants when comparing those with 25(OHD) levels less than 20 ng/mL to those with higher levels. Further, adjustment for vitamin levels accounted for almost half of the excess risk of colorectal cancer seen for black compared with white participants. In a recent systematic review and meta-analysis of 11 observational studies involving 7718 patients with CRC, overall survival was 32% greater when comparing high with low levels of 25(OH)D [85]. Thus, substantial evidence suggests a benefit for vitamin D in reducing CRC incidence and mortality. 

Bladder and kidney cancers. In a meta-analysis of four prospective studies and one case–control study [86], the risk of urinary bladder cancer was 32% higher when comparing low versus high 25(OH)D level (risk ratio = 1.32 (95% confidence interval (CI), 1.15 to 1.89)). In a meta-analysis of two prospective cohort studies and seven nested case-control studies involving 130,609 participants who developed 1815 cases of kidney cancer, the highest 25(OH)D levels were associated with a significant 21% lower incidence (OR = 0.79, (95% CI, 0.69 to 0.91)) of kidney cancer [87]. 

Prostate cancer. In contrast to other cancers, higher 25(OH)D levels have been associated with a modestly higher risk of prostate cancer in prospective studies. A meta-analysis of 19 prospective cohort or nested case-control studies with a total of 35,583 participants and 12,786 prostate cancer cases found that higher 25(OH)D level was associated with increased prostate cancer relative risk = 1.15 (95% CI 1.02 to 1.06) [88]. On the other hand, a meta-analysis of six cohorts of 7648 patients with prostate cancer, for prostate cancer-specific mortality the hazard ratio for high vs. low 25(OH)D was 0.91 (95% CI: 0.88–0.95) for prediagnosis studies and 0.84 (95% CI: 0.58–1.21) for postdiagnosis serum levels [89]. In a case–control study, African-American men with a higher intake of vitamin D had a lower risk of total and aggressive prostate cancer; these associations were not seen in European men [90].

Breast cancer. In a meta-analysis of cohort studies, women with higher versus lower baseline serum levels of 25(OH)D had a barely significant 8% lower incidence of breast cancer [91]; the inverse association was limited to premenopausal women. However, in a pooled analysis of cohort studies with 10,353 cases of breast cancer, standardized serum levels of 25(OH)D were not associated with risk of breast cancer overall or by menopausal status. There was also no statistically significant difference by race (*P* for heterogeneity = 0.90). For the same increment in 25(OH)D levels, the RR was 0.98 (CI, 0.95 to 1.02) in whites (9,579 cases); 1.28 (CI, 0.99 to 1.65) in blacks (290 cases); and 1.13 (CI, 0.76 to 1.68) in Asians (275 cases) [92]. In a cohort of 59,000 African-American women, predicted serum 25(OH)D levels (based on sun exposure, dietary intake, adiposity, and other variables) were inversely associated with risk of breast cancer (1454 cases): risk was 23% higher for the lowest versus the highest quintile [93]. In a recent case–control study among black women, daylight hours spent outdoors per year was inversely associated with lower risk of breast cancer [94].

Total cancer: The VITAL Randomized Trial. In the large VITAL trial [95] participants were randomized to 2000 IU of vitamin D per day and followed for five years. Although vitamin D was interpreted to have no significant overall effect on total cancer incidence, the incidence among African Americans was reduced by 23% (HR = 0.77 (95% CI, 0.59 to 1.01, *p* = 0.06)). Further, after excluding the first two years of follow-up as part of the planned analysis, total cancer mortality was significantly (*p* <0.05) reduced by 25% (HR = 0.75 (95% CI, 0.59 to 0.96)) among all participants. Notably, the inclusion of participants with a relatively high baseline serum 25(OH)D level (mean = 31 ng/mL), many of whom also took supplementary vitamin D, plus the limited duration of follow-up, may have obscured benefits of vitamin D for cancer incidence. In a recent meta-analysis of ten RCTs including VITAL, no benefit of vitamin D supplementation was seen for cancer incidence (6537 cases) [96]. However, cancer mortality was reduced by 13% (95% CI, 4% to 21%) in the five available trials (1591 deaths). In another secondary analysis, there was a significant reduction in advanced cancers (metastatic or fatal) for those randomized to vitamin D compared with placebo [97].

Thus, the findings from randomized trials support vitamin D supplementation for reducing cancer mortality among all participants and cancer incidence among African Americans.

### 2.8. Diabetes Mellitus

In the United States, the age-standardized prevalence of total diabetes is approximately twice as high among non-Hispanic blacks and Hispanics compared to non-Hispanic whites [98]. In a meta-analysis of 28 trials with 3848 participants, vitamin D supplementation reduced HbA1c level by 0.48% (95% CI, 0.18 to 0.79), fasting plasma glucose level by 0.46 mmol/L (95% CI, 0.19 to 0.74), and homeostatic model assessment for insulin resistance (HOMA-IR) level by 0.39 (95% CI, 0.11 to 0.68), in comparison with the control group [99]. Supplemental vitamin D also improved insulin sensitivity in patients with initial low 25(OH)D levels [100]. 

Several RCTs, each reported as negative, have examined how vitamin D affects risk of diabetes among individuals with prediabetes [101,102]. In the Vitamin D and Type 2 Diabetes (D2d) Study among patients with prediabetes (25% of the 2423 participants were African American) [102], those randomized to vitamin D (4000 IU/d) had a nonsignificant 12% (−25 to +4%) lower progression to type 2 diabetes (T2DM) than those receiving placebo [102]. However, in a post hoc analysis among participants with a baseline 25(OH)D level of less than 12 ng/mL (103 participants), progression to diabetes was 62% lower with vitamin D verses placebo (95% CI, 20 to 0.82%). In two other randomized trials, modest and not statistically significant reductions in risk of T2DM were found with vitamin D supplementation [102,103]. If the results for those three randomized trials are combined, the overall reduction in risk is statistically significant (hazard ratio = 0.88; 95% CI, 0.78 to 0.99; *p* = 0.04, unpublished analysis).

Subsequently, an additional secondary analysis of the D2d trial was published [104]. The relationship between intra-trial 25(OH)D levels and incidence of T2DM was determined. The HR for T2DM for an increase of 10 ng/mL in intra-trial 25(OH)D level (*n* = 1074) was 0.75 (95% CI 0.68–0.82) among those assigned to vitamin D and 0.90 (0.80–1.02) among those assigned to placebo. The HRs for T2DM among participants treated with vitamin D who maintained intra-trial 25(OH)D levels of 40–50 (*n* = 319) and ≥50 ng/mL (*n* = 430) were 0.48 (0.29–0.80) and 0.29 (0.17–0.50), respectively, compared with those who maintained a level of 20–30 ng/mL (*n* = 78). In a recent Mendelian randomization (MR) analysis, genetically predicted 25(OHD)D levels, and particularly alleles in genes involved in vitamin D synthesis) were inversely associated with incidence of type 2 diabetes [105].

Thus, the available evidence supports a modest overall benefit of vitamin D in reducing risk of T2DM [102] and possibly a substantial benefit among people with low serum 25(OH)D levels, such as African Americans.

### 2.9. Cardiovascular Disease

Inverse associations have been reported in studies of serum levels of 25(OH)D with risk of cardiovascular disease [106], including analyses specifically among African Americans [107,108,109]. However, no association was seen in MR studies [110] and in the large VITAL trial [95], including in participants with serum levels of 25(OH)D below 20 ng/mL and in African Americans. No association was also seen in a meta-analysis of vitamin D RCTs [111]. 

### 2.10. Alzheimer’s Disease and Dementia and Cognitive Function

Multiple lines of evidence support a role of vitamin D in lowering risk of Alzheimer’s disease (AD) [112]. In a meta-analysis of seven prospective studies and one retrospective cohort study (1953 cases of dementia and 1607 cases of AD), a serum level of 25(OH)D <10 ng/mL was associated with a 31% higher risk of dementia and a 33% higher risk of AD when compared with levels >20 ng/mL [113]. In one prospective study, 30% of participants were African American; higher baseline levels of 25(OH)D were associated with lower rates of cognitive decline but the numbers of were not large enough for race-specific analyses [114].

In MR analyses using the International Genomic of Alzheimer’s Project (IGAP) dataset, risk of AD was found to be lower for individuals with genetic variants predicting higher levels of serum 25(OH)D compared to those without these variants [115]. In the most recent analysis using six such alleles (21,982 cases of Alzheimer’s disease and 41,944 controls), the relative risk per allele was 0.62 (95% confidence interval 0.46 to 0.84) [115]. Together with the data based on serum levels of 25(OH)D, these findings provide substantial evidence that adequate vitamin D will reduce risk of dementia.

### 2.11. Multiple Sclerosis

In ecological studies, consistent with animal models [116], low solar UVB exposure is strongly associated with greater risk of multiple sclerosis (MS) [117] in whites, African Americans, and Hispanic Americans [118]. The inverse association between UVB exposure and MS also was seen in studies of individuals [119,120]. Solar UVB exposure in winter appears especially important.

In cohort studies, serum levels of 25(OH)D have been inversely associated with risk of MS [121]. In U.S. military recruits, levels greater than 40 ng/mL were associated with the lowest risk of MS, a level few African Americans attained [121]. In a Swedish study, 25(OH)D levels in the highest quintile were associated with a 32% lower incidence of MS [122]. Low levels of 25(OH)D in neonatal blood spots were strongly associated with MS later in life [123], supporting the importance of maternal vitamin D status during pregnancy. In a large cohort of women, use of vitamin D supplements greater than or equal to 400 IU/day was associated with lower risk of MS, but intake from diet, which rarely exceeds 400 IU/day, was not [124]. A cohort study conducted in southern California included a modest number of Black, Hispanic, and White participants with MS and matched controls. An inverse association between serum 25(OH)D level and incidence of MS was seen in Whites, but not among Blacks or Hispanics [118], but in all three groups a careful assessment of lifetime solar exposure was inversely associated with risk of MS. The authors suggested that something about solar exposure independent of vitamin D may be protective for MS, but an alternative explanation could be that their lifetime solar exposure assessment provided a better indication of long term vitamin D status (which would have been correlated with solar exposure over this period) than a single blood measurement collected in midlife.

A review of vitamin D supplementation in MS found little benefit even at high vitamin D doses [125]. The reasons for lack of benefit of vitamin D supplement suggested by the authors included the number of participants being too low, the length of the trial too short, baseline serum 25(OH)D levels too high, and other treatments being administered reducing the potential of vitamin D to help.

In a MR analysis of two large cohorts including 7391 cases of MS and 14,777 controls, a genetic risk score comprised of three alleles known to be associated with higher plasma 25(OH)D predicted levels was associated with lower risk of MS [126]. In the meta-analyses of these cohorts, the relative risk per allele was 0.85 (95% CI, 0.76 to 0.94, *p* = 0.003). This result, in combination with the other extensive observational study evidence, supports a protective role of adequate vitamin D intake for incidence of MS.

### 2.12. Acute Respiratory Tract Infections and COVID-19

Substantial evidence indicates that higher serum 25(OH)D levels can reduce the risk or severity of acute respiratory tract infections, possibly including COVID-19. Potential mechanisms include role of vitamin D in innate and acquired immunity [127,128]. This relation was suggested by observations that the seasonal increase in influenza infections corresponds with lower solar UVB doses and 25(OH)D levels [129] and that in the 1918-1919 influenza pandemic the case-fatality rates were much lower in the southwestern U.S. states than in the northeastern states [130]. In a meta-analysis of 25 RCTs involving 10933 participants, vitamin D supplementation (daily or weekly) reduced risk of acute respiratory tract infections by 19% [131]. For participants with baseline 25(OH)D level <10 ng/mL, the reduction was 70%. In a post hoc analysis of an RCT conducted among 208 postmenopausal African-American women living in New York, supplementation with 1000 or 2000 IU/day of vitamin D_3_ compared to a placebo significantly reduced rates of influenza and colds [132].

Adequate vitamin D supplementation has also been hypothesized to decrease incidence and death from COVID-19; in addition to reducing viral replication, vitamin D may limit excess production of pro-inflammatory cytokines underlying the “cytokine storm” that damages the lungs and other organs [130,133]. Incidence and mortality of COVID have been far higher in African Americans than in European Americans [134]; after adjustment for age, African Americans are 4.5 times more likely to die from COVID-19 than European Americans [135]. Much of this could be due to more crowded housing, riskier jobs, dependence on public transportation, and higher prevalence of existing cardiometabolic conditions, but the high prevalence of vitamin D deficiency may also contribute. 

Among patients with COVID-19, the disease resulting from SARS-CoV-2 infection followed by a dysregulated immune response, low levels of 25(OH)D at time of diagnosis have been associated with more severe illness [136,137,138]. Reverse causation cannot be excluded because serum 25(OH)D level decreases in response to acute inflammatory disease [139,140], but serum 25(OH)D levels have been associated with SARS-CoV-2 virus positivity using seasonally-adjusted 25(OH)D levels from the preceding 12 months [141,142].

The strong suggestion of benefits of vitamin D supplementation for preventing or treating COVID-19 has encouraged the initiation of supplementation trials [143]. In a non-randomized intervention study conducted in Spain among hospitalized patients hospitalized for COVID-19 [144], high doses of vitamin D (as 25(OH) D_3_) were administered in combination with standard care; only 1/50 required admission to the intensive care unit compared to 13/26 comparable control patients. An RCT conducted in India involving 40 SARS-CoV-2 positive patients with serum 25(OH)D with mean values near 9 ng/mL were randomized into high-dose vitamin D treatment (*n* = 16) and control (*n* = 24) groups [145]. Ten (63%) participants in the intervention group and five (21%) participants in the control arm (*p* < 0.02) became SARS-CoV-2 RNA negative. According to the registry of clinical trials [146] as of 30 December 2020, there were at least 35 RCTs registered examining the role of vitamin D supplementation in prevention or treatment of COVID-19. In these trials, it will be important to distinguish among those with low versus adequate vitamin D status at baseline.

### 2.13. Asthma Exacerbations

A combined analysis of two RCTs conducted with pregnant women found that vitamin D supplementation (2400 IU/d and 4000 IU/d) reduced risk of asthma/recurrent wheeze from 0–3 years by 24% (aOR = 0.74 (95% CI, 0.57 to 0.96)) [147]. The effect was strongest for those with baseline 25(OH)D level ≥30 ng/mL (aOR = 0.54 (95% CI, 0.33 to 0.88)). A secondary analysis of the 4000 IU/d vitamin D RCT found that there was no difference with respect to race for African American vs. non-African American [148]. In a meta-analysis of individual participant data from seven RCTs with high-quality evidence, vitamin D supplementation reduced by 26% the risk of asthma exacerbation that required treatment with systemic corticosteroids [149]. The reduction was 67% for individuals with initial 25(OH)D level <10 ng/mL (92 participants). Thus, there is good evidence that raising serum 25(OH)D levels reduces risk of asthma or its exacerbation.

### 2.14. All-Cause Mortality

**Figure 3:**
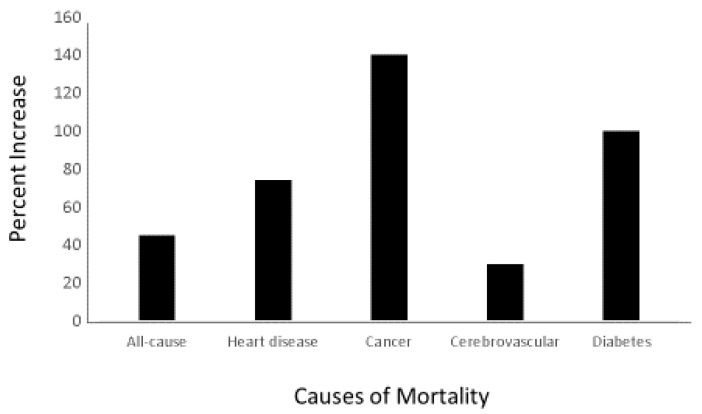
Percentage Increase in Cause-Specific Mortality for Black compared to White Americans, Ages 50–64 years—United States, 2015 [150].

A comparison of death rates for African Americans with those of European Americans shows a large disparity for many diseases [150]. For example, the disparity in death rates from all causes for people aged 50 to 64 years is 45% higher in African American than in European Americans (Figure 3). Differences in multiple factors, such as hypertension, obesity, diet, income, education, and lower access to medical care, may contribute to some of those disparities, but they do not fully explain the differences [151,152,153,154,155].

In a meta-analysis (~29,000 subjects from five Northern European countries), serum levels of 25(OH)D below 32 ng/mL were associated with the highest mortality [156]. In another meta-analysis of 32 studies, mortality for those in the lowest (<9 ng/mL) was about double compared with those with the highest serum 25(OH)D level. The lowest mortality was reached near 40 ng/mL and plateaued above this level [157]. Most of those analyses accounted for potentially confounding variables such as adiposity, physical activity, and smoking, but some residual confounding could not be excluded. 

More direct evidence for causality comes from a MR analysis documenting an association between genetically determined serum 25(OH)D levels and both total and cancer-specific mortality [110]. Although in a MR analysis from the UK Biobank genetically determined 25(OH)D level was not associated with all-cause mortality rate [158], that study was underpowered according to the authors, especially given that the association appears nonlinear. 

**Figure 4:**
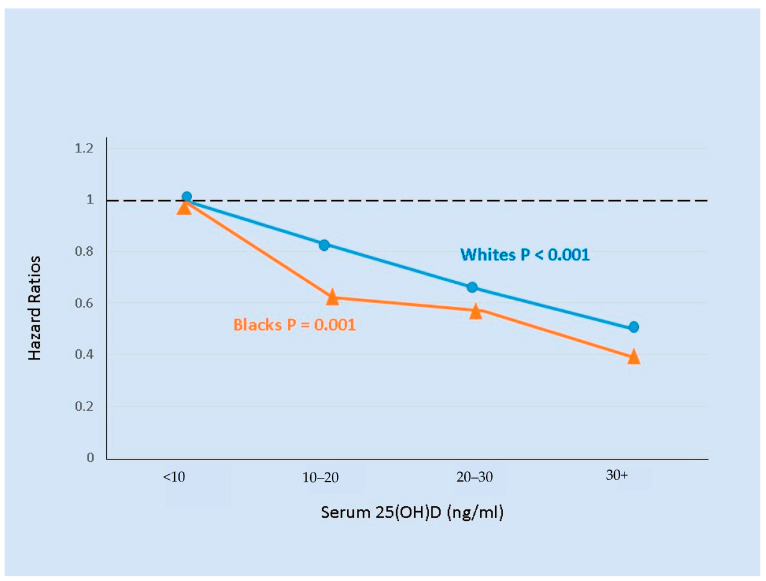
Serum 25(OH)D levels and all-cause mortality for elderly Black (*n* = 1023) and White (*n* = 1615) men and women followed for up to 8.5 years [159]. Hazard ratios with < 10 ng/mL serum 25(OH)D as the reference were adjusted for other predictors of mortality.

A US cohort of 3075 adults aged 70–79 years of age was followed for 8.5 years [159]. Although the prevalence of vitamin D deficiency was much higher in Blacks than in Whites, lower baseline serum levels of 25(OH)D were similarly associated with higher mortality in both Black and White participants (Figure 4). Because of the large Black/White difference in vitamin D status, 25(OH)D levels below 30 ng/mL statistically accounted for 38% of mortality in Blacks and 11% in Whites. In a multivariate model without 25(OH)D levels, Blacks had 22% higher mortality than Whites, but after inclusion of 25(OH)D in the model the excess mortality in Blacks was only 9% and not statistically significant. 

Similar findings were seen in a nested case-control analysis from a cohort of largely African-American participants, in which 1852 cohort members who died were matched to a similar number of participants who remained alive. Using baseline serum samples, the multivariate OR for death for those in lowest quartile compared with the highest quartile was 1.60 (95% confidence interval (CI): 1.20, 2.14, *p*_trend_ = 0.003) for African Americans and 2.11 (95% CI: 1.39, 3.21, *p*_trend_ < 0.001) for non-African Americans; the adjusted mortality rate became flat above approximately 30 ng/mL for both groups [160]. In the VITAL trial, no significant effect of vitamin D supplements on total mortality was seen in black or white participants, but the study was limited by duration and relatively high baseline levels [95].

Thus, there is good evidence from observational studies that all-cause mortality rate is inversely correlated with serum 25(OH)D concentrations up to about 30 to 40 ng/mL and then the association becomes flat.

## 3. Discussion

This review calls attention to the health-related consequences of low 25(OH)D levels for people with dark skin living at high northern latitudes, in both annual average and winter or summer [31,161]. Serum 25(OH)D levels are much lower among people with dark skin than among those with light skin living at similar latitudes. The prevalence of a serious deficiency value of 10 ng/mL (or less) is particularly high among African Americans. Regardless of skin color, low 25(OH)D levels are associated with higher incidence or poorer outcomes for many diseases. Evidence is particularly strong for several complications of pregnancy, multiple sclerosis, dementia, type 2 diabetes, colorectal cancer, total cancer mortality, and acute respiratory tract infections. For several of these diseases, causality is supported by either RCTs (such as for cancer mortality [95], diabetes mellitus [102], and acute respiratory tract infections [131]) or by the combination of prospective cohort and MR studies (such as for MS and dementia). Even if not all those relationships are ultimately determined to be causal, the consequences of vitamin D deficiency on the remaining diseases are important. On the basis of that evidence, vitamin D deficiency is highly likely to contribute to disparities in health status between people with dark and light skin at high latitudes. Hopefully, this review, by assembling the latest information on vitamin D for many health outcomes, will motivate physicians and patients to consider improving vitamin D status as an efficient way to improve health regardless of skin type.

Our study contrasts with the interpretations of several recent major RCTs on vitamin D supplementation [95,102] which reported no benefit of vitamin D supplementation. Although RCTs of vitamin D supplementation can be useful or definitive if a clear effect is seen, they also can be misinterpreted or misleading if no statistically significant effect is seen for various reasons, such as that the baseline and achieved 25(OH)D levels were either not measured or not considered in designing the trial [162,163]. Therefore, important benefits would be missed if many participants had levels high enough before randomization. For ethical or practical reasons, many RCTs do not focus on participants with vitamin D deficiency [95,102,164] who would be the people who would benefit most from supplementation. Also, some trials permit or encourage all participants to take additional vitamin D (400 to 800 IU/d), thereby reducing the risk of disease in the control group [95]. For some trial outcomes, especially cancer incidence, supplementation for long periods may be needed, but the effort is complicated by declining adherence. Our reasons for reaching a different conclusion include consideration of a broader literature, including important recent studies, and of secondary analyses for subpopulations, such as African Americans, most likely to benefit [165].

Observational cohort studies can circumvent some of those problems, but some residual confounding may be hard to exclude. Community-based observational studies in which participants take a vitamin D dose of their choice, have 25(OH)D levels measured semiannually, and report any changes in health status such as those conducted by GrassrootsHealth.net, e.g., [166], can play a role. No single type of study will provide the best evidence for all hypotheses, and the greatest insights will come from a thoughtful combination of research strategies. Those studies should especially include people with dark skin, in particular African Americans.

### Preventing Vitamin D Deficiency

Few foods, mainly fish and fish liver, have substantial amounts of vitamin D, which is primarily synthesized in the skin. Therefore, low 25(OH)D levels are largely determined by melanin levels and contemporary lifestyles (including getting little sun exposure by staying indoors, covering the body, and using sunscreen extensively) and excess body fat [167]. For people with dark skin, especially if living at northern latitudes and in winter, typical sun exposure will usually not be adequate to prevent deficiency. Notably, while leisure-time sun exposure contributes to serum levels of 25(OH)D in European Americans, it does so minimally in African Americans [168]. Vitamin D supplementation can effectively prevent deficiency. Fortifying milk with vitamin D has prevented rickets in children, but the amount of vitamin D (100 IU per 8 oz [240 mL]) would have only small effects on serum levels in adults. In addition, milk consumption has decreased over time and lactose intolerance is common, especially among African Americans [169]. Thus, intakes may need to be increased primarily by fortifying additional foods, including non-dairy, or use of supplements; issues of dose and frequency of administration suggest that levels of at least 30 ng/mL would be a reasonable target. Notably, while solar UVB exposure is an important source of vitamin D, African Americans have high prevalence of 25(OH)D levels below 30 ng/mL in both summer (88%) and winter (93%), which contrasts for European Americans (61% in winter and 49% in summer) and Hispanics (86% in winter and 57% in summer) [161].

One strategy would be to screen routinely and to supplement people with low serum levels, but ideally a safe dose could be identified that yields near-optimal serum levels for almost everyone. A level of 20 ng/mL or higher was considered sufficient by an IOM committee in 2011 [36]; however, levels between 20 and 30 ng/mL have also been associated with lower risks of colorectal cancer [170], total mortality [157], dementia [113], multiple sclerosis [122] and bone mineral density [49,171].

Other researchers have suggested that 40 to 60 ng/mL is optimal based on results of small observational studies with participants taking high vitamin D_3_ doses [172,173,174]. Three-quarters of African Americans not already taking supplements have levels that do not ensure adequacy even by the IOM definition (20 ng/mL), and 96% have levels below 30 ng/mL. Notably, for African Americans living in Boston, 4000 IU/day was required to achieve serum levels of 30 ng/mL [175]. Similarly, among men with early prostate cancer, serum levels of 25(OH)D were much lower in African Americans compared with European Americans, but after supplementation of 4000 IU/day for one year, levels increased in both groups and were nearly identical [176]. In 2012, vitamin D supplements were used by only 12% of African Americans and 22% of European Americans [177]. Noting the between-person variation in response to the same dose of vitamin D, some have suggested the desirability of monitoring indicators of biological function [178]. While this deserves consideration, it would add greatly to costs, and the specific variables to monitor are not clear at this time [179].

Vitamin D supplementation up to 4000 IU of vitamin D daily was considered to be safe by the 2011 IOM review. Further assurance comes from the trial using 4000 IU/day for 2.5 years [102]. An intake of 4000 IU/day has been used without adverse effects during pregnancy [61,67]. Higher intakes may also be safe [170,180] and warrant further study. At very high doses, such as in accidental exposures, vitamin D can produce death, neurological symptoms and serious damage (e.g., 1 million IU/day for several weeks, although the damage can sometimes be reversed [181]); at less extreme doses, a primary concern has been hypercalciuria and kidney stones [182], although any excess risk of kidney stones appears to be minimal when taking vitamin D supplements up to 4000 IU per day [183].

## 4. Conclusions

Together, ecological studies, prospective cohort studies based on blood levels, MR studies, and randomized trials provide moderate-to-strong evidence that low levels of 25(OH)D have many adverse health consequences. In addition, the fact that the vitamin D hormone, calcitriol, controls a considerable percentage of the human genome [1] indicates that it must be of huge general importance for health.

Much evidence is at present derived from studies of people of European descent. However, the benefits of supplementation for most health outcomes (other than skeletal effects) probably apply to all groups but are likely to be greatest for people with dark skin living at higher latitudes, such as African Americans, as well as most people in winter and those spending little time in the sun during summers. Many of the health disparities we discuss also have a basis in income inequality, poorer education and employment opportunities, poor housing, food insecurity, and other social inequalities; efforts to improve 25(OH)D levels will not lessen the need to address these factors but should improve health outcomes. While further research is needed to identify the optimal strategy for vitamin D supplementation and fortification, no reason exists to delay addressing vitamin D deficiency among populations with high prevalence of deficiency such as African Americans. The potential benefits promise to be large, and much evidence indicates that the risks of supplementation up to 4000 IU per day vitamin D are minimal.

## Figures and Tables

**Table 1:** Pregnancy and birth outcomes as a function of ethnicity.

Outcome	Ethnicity (%)			Ratio		Ref.
	Black	Hispanic	White	Black/White	Hispanic/White	
Cesarean delivery	35.9	31.7	30.9	1.17	1.03	[54]
In-hospital mother death rate	0.21	0.05	0.05	4.23	0.98	[55]
Preeclampsia,	9.8	7.7	6.7	1.50	1.10	[56]
Low birth weight	13.7	7.3	7.0	2.00	1.04	[54]
Preterm birth	13.9	9.6	9.1	1.50	1.06	[54]
Small for gestational age	10.8	6.5	5.7	1.91	1.14	[57]

**Table 2:** Incidence and mortality rates for select cancers in the U.S. for males and females, 2008–2012 [74].

Sex and Cancer Type	Incidence *			Mortality **		
	Black	White	Black/White Ratio	Black	White	Black/White Ratio
**Male**						
Prostate	208.7	123.0	1.70	47.2	19.9	2.38
Lung	93.4	79.3	1.18	74.9	62.2	1.20
Colorectal	60.3	47.4	1.27	27.6	18.2	1.52
Kidney	24.2	21.8	1.11	5.7	5.9	0.97
Liver	16.5	9.3	1.77	12.8	7.6	1.69
Stomach	15.1	7.8	1.93	9.4	3.6	2.58
**Female**						
Breast	124.3	128.1	0.97	31.0	21.9	1.42
Lung	51.4	58.7	0.87	36.7	41.4	0.89
Colorectal	44.1	36.2	1.22	18.2	12.9	1.41
Kidney	13.0	11.3	1.15	2.6	2.3	1.13
Stomach	8.0	4.3	2.30	4.5	1.8	2.48
Liver	4.8	3.2	1.52	4.4	3.1	1.43

* Age-adjusted cases/100,000/yr; ** Age-adjusted deaths/100,000/yr.

## Appendix A

Many types of epidemiological study are used to determine the extent to which vitamin D affects health outcomes. The strengths and weaknesses of the types of study used to determine the relationship between vitamin D and health outcomes are outlined in Table A1.

**Table A1:** Comparison of epidemiological approaches to determine relationships between vitamin D and health outcomes.

Approach	Method	Strengths	Weaknesses
Ecological, geographical	Compare health outcomes with indices of solar UVB doses and other risk-modifying factors	Can include large numbers of participants	Subject to confounding factors; indices used may not apply to those with health outcomes
Observational, prospective	Enroll participants, draw blood, obtain information, follow for a long period.	25(OH)D has inputs from solar UVB, diet, and supplements.	25(OH)D changes with time including season so effect decreases as follow-up time increases; control of confounding may not be complete.
Observational, case-control	Measure 25(OH)D near time of diagnosis, match with controls.	Appropriate when health outcome is affected by recent 25(OH)D or 25(OH)D changes little over time.	Disease status may affect 25(OH)D; control choice may be biased.
Observational, cross-sectional	Measure 25(OH)D and health status of a representative number of a population	Many health outcomes can be studied.	25(OH)D may not be similar to that prior to health outcome; health status may affect 25(OH)D.
Non-randomized vitamin D supplementation	Enroll participants, measure parameters, instruct.	High vitamin D doses can be used;	Confounding by self-selected use of supplements
RCT	Enroll participants, randomize to vitamin D or placebo supplementation, follow.	Effects found are likely due to vitamin D.	False negatives are possible because enrollees often have high 25(OH)D or may be given low vitamin D doses; compliance issues; other sources of vitamin D occur. Initiation may be after onset of disease and duration may be too short to have an effect.
MR	Measure alleles of genes that affect 25(OH)D levels.	Independent of many factors including actual 25(OH)D.	The alleles may not reflection biological activity, and confounding is still possible. Can be misleading when relationship of vitamin D is nonlinear, and statistical power can be low.

MR—Mendelian randomization; RCT—Randomized controlled trial.
